# Mechanical Performance and Surface Roughness of Lithium Disilicate and Zirconia-Reinforced Lithium Silicate Ceramics Before and After Exposure to Acidic Challenge

**DOI:** 10.3390/dj13030117

**Published:** 2025-03-06

**Authors:** Ahmed Elsherbini, Salma M. Fathy, Walid Al-Zordk, Mutlu Özcan, Amal A. Sakrana

**Affiliations:** 1 Department of Oral-Maxillofacial Surgery, Dentistry and Orthodontics, Tissue Engineering Division, The University of Tokyo, Tokyo 113-0033, Japan; aelshal-omfs@g.ecc.u-tokyo.ac.jp; 2 Dental Biomaterials Department, Faculty of Oral and Dental Medicine, Zagazig University, Zagazig 44519, Egypt; salmamf@zu.edu.eg; 3 Department of Fixed Prosthodontics, Faculty of Dentistry, Mansoura University, Mansoura 35516, Egypt; drwalid@mans.edu.eg; 4 University of Zurich, Center for Dental Medicine, Clinic for Masticatory Disorders and Dental Biomaterials, 8032 Zurich, Switzerland; mutlu.ozcan@zzm.uzh.ch

**Keywords:** acidic challenges, advanced lithium silicate, dental materials, flexural strength, lithium silicate-based ceramics, surface properties, zirconia-reinforced lithium silicate

## Abstract

**Background/Objectives**: Fabrication of ceramic restorations with higher performance, biocompatibility, and mechanical durability, as well as excellent optical properties, is challenging. Therefore, this study was designed to investigate the mechanical properties and surface roughness of different glass ceramics manufactured with CAD/CAM and pressed techniques before and after exposure to simulated gastric acidic challenge. **Methods**: Lithium disilicate-based (LDS) ceramic, advanced lithium disilicate (ALDS), and zirconia-reinforced lithium silicate (ZLSC), were manufactured with two techniques. Disc-shaped (N = 336) specimens were used to test the biaxial flexural strength (BFS), while surface microhardness and surface roughness were evaluated before and after exposure to hydrochloric acid (34–37% concentration and 1.2 pH for 24 h). The results were statistically evaluated using two- and one-way ANOVA, Tukey’s post-hoc and Student’s *t*-test. **Results**: Both CAD and pressed types of LDS ceramic IPS e.max, showed significantly highest BFS and microhardness before and after acidic challenge (*p* = 0.01 and <0.01, respectively). ALDS and ZLSC showed significantly lower roughness before acidic aging, while all groups showed no significant difference after aging. **Conclusion**: Pressed groups showed better mechanical performance than CAD groups. LSD (IPS e.max types) continuously showed a better mechanical performance than modified LDS. ALDS and ZLSC showed the best performance in terms of surface smoothness, but after acidic exposure, no significant difference was observed compared to the other groups.

## 1. Introduction

Ceramic restorations are gaining more popularity and showing improvements in their performance as a result of their high biocompatibility, mechanical durability and excellent esthetics [1,2]. Although they are reported as inert materials, they are exposed to high acidic challenges in the oral environment. These challenges include the ingestion of citrous fruits or beverages, the inhalation of industrial acidic fumes, and gastric acid reflux as in the case of bulimia and anorexia nervosa [3]. The latter aspects are considered as extrinsic factors affecting the mechanical performance of ceramics [4]. These acidic challenges are the main causes of erosive tooth wear, which is considered of high global concern [5]. This could, most probably, endanger ceramic restorative materials, like glass-ceramics, which are used for treating erosive tooth wear resulting from even more acidic challenges intra-orally [6].

Other intrinsic factors related to ceramic compositions, microstructure, surface finish and glaze could also play a role in ceramic response to acidic challenges in the oral cavity [4]. A recent evidence-based study revealed that immersion or rinsing with different acidic agents caused a significant increase in surface roughness and a decrease in surface microhardness, which mostly endangered glass ceramics and indirect composites compared to polymer-infiltrated ceramic [6]. The degradation in surface properties, like roughness and microhardness, are critical factors affecting the long-term success and aesthetic properties of ceramic restorations. Coarse surfaces could adversely affect tooth wear, enhancing both plaque accumulation and stress concentrations on or within ceramic surfaces. Furthermore, degradation in surface microhardness could aggravate the wear resistance and flexural strength of ceramics [7]. In addition to surface properties, acidic challenges may also affect the brittle behavior of ceramics, making them more prone to fracture [8].

Furthermore, variations in the processing of lithium-based glass ceramics may affect their distinctive inner microstructures. The latter could, in turn, affect the mechanical performance and thus the clinical performance of such ceramics [9,10]. These lithium-based glass ceramics could be processed through the milling of fully crystallized (one-step) or partially crystallized (two-steps) ceramic blocks or those ingots intended for injection-molding techniques—either hot pressed or lost wax techniques [11]. Additionally, uncontrolled crystallization might occur throughout the firing procedures of these glass-ceramics, resulting in a change in the thermal behavior and thus the clinical survival of the materials [12].

The diverse chemical and structural compositions of available CAD-CAM materials may result in different responses to erosive fluids, with a potential change in the surface roughness [13]. Erosive intraoral conditions could cause chemical degradation and surface ion release, which may cause alterations in the ceramic surface microstructure and topography [14,15]. Disilicate glass ceramics showed significant weight loss with reductions in the surface roughness of some groups compared to zirconia generations after gastric acid immersion [16]. It was recommended to undertake further laboratory studies on ceramic blocks, especially those manufactured with CAD/CAM techniques, which, in turn, will improve knowledge concerning the reliability and the clinical relevance of the findings [17].

Dental manufacturers often classify and promote their products with reference to their resistance to chemical degradation and mechanical strength characteristics, such as flexural strength, fracture toughness, critical crack sizes and fatigue resistance [18]. As such, the objectives of the current study were to evaluate the impact of acid challenge on the properties of some types of commercially available lithium silicate and zirconia-toughened glass ceramics, manufactured with two techniques. The null hypotheses of the current study were that:

1. There would be no significant impact of glass ceramic composition and manufacturing techniques, pressed or CAD/CAM, on the surface micro-hardness, roughness, or biaxial flexural strength.
2. There would be no significant difference between the tested properties of all ceramic groups, pressed and CAD/CAM, before and after exposure to acidic challenge.

## 2. Materials and Methods

The materials used in the current study, along with their compositions, are mentioned in Table 1.

### 2.1. Specimen Preparation and Glass Ceramic Processing:

PS Power software 3.1, developed in Vanderbilt Biostatistics (Vanderbilt university Nashville, TN, USA), was used for sample size calculation. For the study design plan, it was found that in previous studies on mechanical testing, the BFS results were normally distributed with a standard deviation of 53.98. If the true difference between experimental and 100 reference groups’ means was 137.01 MPa, the sample size would be set at n = 5 for each group in BFS [21]. Similarly, regarding previous results of surface microhardness [21], they were normally distributed, and they showed a standard deviation of 0.34. If the true difference in experimental and reference groups means were 1.34 GPa, the sample size would be set at n = 10 for each group. The same **n** values of ten specimens were obtained for surface roughness (Ra) when the previously obtained standard deviation was 0.47 and the true difference between means of experimental and reference groups was 0.82 μm [22].

a. Fabrication of pressable glass ceramics:

Here, 168 disc-shaped specimens with dimensions of 10 mm in diameter and 1.2 mm in thickness were 3D digitally designed with the help of a specific software exocad (exocad Dental CAD; exocad GmbH, Darmstadt, Germany 2010–2022). The standard tessellation language (STL) file created was used to print the previously mentioned disc from a wax material using a 3D printer machine (Anycubic Photon Mono X, Shenzhen, China). After printing, the wax discs were washed and cured using the wash and cure machine (Anycubic wash & cure plus, Anycubic technology Co., Hong Kong).

Afterwards, these wax pattern discs were sprued (using 3 mm-long and 2.5 mm-diameter sprue) and invested with an investment prepared according to the manufacturer’s instructions. Investment rings were placed in a furnace (Vulcan S-550 Autonics furnace, Melville, NY, USA) at 850 °C for 60 min for wax burnout. Then, pressable ceramic ingots were placed on investment rings with the plunger in position. They were placed in a ceramic heating furnace (Programat EP 3010 press, Ivoclar Vivadent, Ellwangen, Germany) and the firing program was selected and activated according to each type of glass ceramic. After complete investment cooling, the pressed ceramic specimens were de-invested, sandblasted (Vario jet, Renfert, Hilzingen, Germany) and ultrasonically cleaned by placing them in IPS e.max Invex liquid (Ivoclar Vivadent, Ellwangen, Germany) for 15 min. Afterwards, all samples were polished utilizing 500-grit silicon carbide discs for 60 s at a speed of 200 rpm in the presence of a water-cooling system though a polishing machine (MetaServ 250 Grinder-Polisher with Vector Power Head, Buehler, IL, USA). Specimen glazing was performed according to the manufacturer’s specifications. Table 2 shows all specification parameters for pressing and glazing steps. Subsequently, half the pressed ceramic disks were immersed from the glazed side in hydrochloric (HCl) acid (34–37% concentration and 1.2 pH) solution (PGI chemicals, Runcorn, UK) for 24 h [4].

Afterwards, the specimens were washed with distilled water for 10 s and dried in a furnace (TR 60, Nabertherm, Lilienthal, Germany) at 35 °C for 24 h before testing. Half of the other specimens, which acted as a control group, were ultrasonically cleaned with distilled water for 10 min, then cleaned and dried as previously mentioned. The parameters for processing pressed specimens are mentioned in Table 1. All pressable ingots were of high translucency (HT).

b. Fabrication of CAD/CAM glass ceramics

An equal number of disc-shaped specimens with the same dimensions as previously mentioned for the pressable glass ceramic section were fabricated. The previously designed STL file of the disc-shaped specimens was used to mill the required number of specimens using a milling machine (inLab IMES-ICORE 150i PRO, GmbH, Hessen, Germany). All specimens were finished and polished with the same protocol as applied for pressed ceramic specimens. Subsequently, specimens that needed further crystallization were crystallized. Then, all specimens were glazed according to the manufacturer’s instructions using the furnace previously used for the pressed ceramics. The parameters for all machinable specimens are mentioned in Table 3. All CAD/CAM blocks were of HT.

### 2.2. Mechanical Testing

a. Biaxial flexural strength (BFS):

Here, 96 specimens (disc-shaped; 10 × 1.2 mm diameter and thickness, respectively) were used to evaluate the biaxial flexural strength (BFS) for all groups before and after exposure to acidic challenge, with each group containing 8 specimens, including 3 to be used for surface topography examination. The test was performed according ISO standard 6872:2024 [23]. Specimens were placed on 3 stainless steel balls with a diameter (ø) = 3.2 mm, which were positioned 120° apart from each other within a circle (ø = 10 mm). The specimens were loaded at their center using a piston (ø = 1.6 mm) attached to a universal tester (3345, Instron, Canton, MA, USA) with a cross-head speed 1 mm/min. The glazed surface exposed to acidic challenge was set facing downward during testing. The force at fracture point was recorded using the computer software, and BFS was calculated using the following formula:

𝜎 = −0.2387*P*(*X* − *Y*)∕*d*^2^,

*X* = (1 + *v*) In (*r*2∕*r*3)^2^+ [(1 − *v*)∕2] (*r*2∕*r*3)^2^,

*Y* = (1 + *v*) [1 + In (*r*1∕*r*3)^2^] + (1 − *v*) (*r*1∕*r*3)^2^,

with *σ* being biaxial flexural strength (MPa), *P* the force at failure (N), *d* the thickness of the specimen (mm), *v* the Poisson’s ratio, r1 the radius of the support circle (mm), *r*2 the radius of the loaded area (mm), and *r*3 the radius of the specimen (mm).

b. Vicker’s surface microhardness:

Here, 120 specimens (disc-shaped; 10 × 1.2 mm diameter and thickness, respectively) were used to evaluate surface microhardness for all tested groups before and after exposure to acidic challenge. A load of 300 g (HV 0.3) was applied for 10 s dwell time to make three indentations on each specimen’s surface, away from the specimen margins (2 mm all over the margins). The load was removed, and then the resulting indentation was assessed with the magnifying eye piece. The two diagonal impressions were measured, usually to the nearest 0.1 μm, with a micrometer, and then averaged. The indentations were made using a Vicker’s device (Tukon 1102 Wolson microhardness tester, Buehler, Germany). The Vicker’s hardness (HVN) was calculated using

HV = 1854.4 L/d^2,^

where the load L is in gf, and the average diagonal d is in μm (produces hardness number units of gf/μm^2^).

### 2.3. Surface Roughness

For surface roughness measurements, 120 specimens (disc-shaped; 10 × 1.2 mm diameter and thickness, respectively) were used to evaluate surface roughness (Ra) for all tested groups before and after exposure to acidic challenge. Each specimen was fitted to the specimen holder in which the surface to be measured was set in a horizontal direction. The holder was then moved in a vertical direction up to a level where the specimen’s top surface nearly touched the profilometer measuring tip. Ra calibration was performed at a measuring distance of up to 8 mm, with a measuring speed of 0.5 mm/s and returning speed of 1 mm/s, as well as a measuring force of 0.75 mN. The stylus profile tip radius was 2 μm and the tip angle was 60° in contact mode. For each specimen, five Ra readings were taken away from the specimen margins (2 mm all over the margins) using a profilometer (Surftest SJ 210, Mitutoyo, Kanaqawa, Japan).

### 2.4. Scanning Electron Microscopy Analysis (SEM)

Three specimens were selected randomly from each BFS group before BFS testing to be gold-spattered and then examined under SEM (SEM; JEOL Ltd., Peabody, MA, USA) under a high voltage of 30 kV and magnification ×1000. An examination was performed to evaluate the surface topography of all groups before and after acid challenge exposure by HCl. For the microstructural crystal morphology evaluation, three specimens were collected randomly form each group after BFS. Specimens were etched using hydrofluoric acid 9.5% for 30 s and then gold-sputtered to be examined with the SEM device under ×3000 magnification.

### 2.5. Statistical Analysis

Statistical analysis was performed using SPSS 16.0 (SPSS, Chicago, IL, USA) for Windows. The results were first analyzed for normality using Shapiro–Wilk followed by two-way ANOVA and Tukey’s post-hoc for all mechanical testing before and after acidic challenge. An independent Student *t*-test was performed to evaluate the statistical difference for groups after exposure to acid challenge.

## 3. Results

### 3.1. Mechanical Testing Results

- Biaxial flexural strength (BFS):

Two-way ANOVA showed the statistically significant impact of only material composition on BFS (*p*-value = 0.002 and 0.001 before and after acidic challenge, respectively), while there was no statistically significant impact of either manufacturing technique or the interaction of the two factors between before and after (Table 4 and Table 5). IPS e.max types showed significantly higher BFS, with no significant difference between them, while GC-LiSi-CAD showed the lowest statistically significant BFS value, all for cases, before and after acidic aging. Although there was a decrease in all groups in the BFS after acidic aging, this decrease was not statistically significant. Table 6 shows all *p*-values for groups after acidic aging.

b. Vicker’s surface microhardness:

The two-way ANOVA showed the statistically significant impact of both variables, material composition and manufacturing process, in addition to the interaction between them, on surface microhardness before and after aging (Table 4 and Table 5). IPS e.max Press followed by IPS e.max CAD showed the (statistically significantly—*p* = 0.000) highest surface hardness before acidic exposure, while ZLSC (Celtra Duo Press) showed the significantly highest surface hardness after acidic exposure (*p* = 0.000). GC-LiSi-CAD showed the lowest statistically significant surface hardness before acidic exposure. All surface microhardness values showed statistically significant decreases after acidic aging (Table 7).

### 3.2. Surface Roughness

Two-way ANOVA showed the statistically significant impact of both manufacturing technique and the interaction between manufacturing technique and composition on surface roughness (Ra) before aging, while there was no statistically significant difference caused by any of the variables or interactions between them in the Ra values after acidic aging (Table 4 and Table 5). Celtra Duo Press and Cerec-Tessera-CAD showed the significantly lowest Ra before acidic exposure, with no significant difference between them. On the other hand, GC-LiSi-CAD showed the highest Ra before aging with no statistically significant difference in its pressed type or both IPS e.max types. After acidic exposure, all groups showed no statistically significant Ra values among them (*p*-value = 0.119). After acidic challenge, Celtra Duo Press and Cerec-Tessera-CAD showed statistically significant increases in Ra (*p*-value = 0.006 and 0.005, respectively), while all remaining groups showed decreases in Ra with no statistically significant difference from their control before acidic exposure (Table 8).

### 3.3. Scanning Electron Microscope (SEM) Analysis

**Figure 1:**
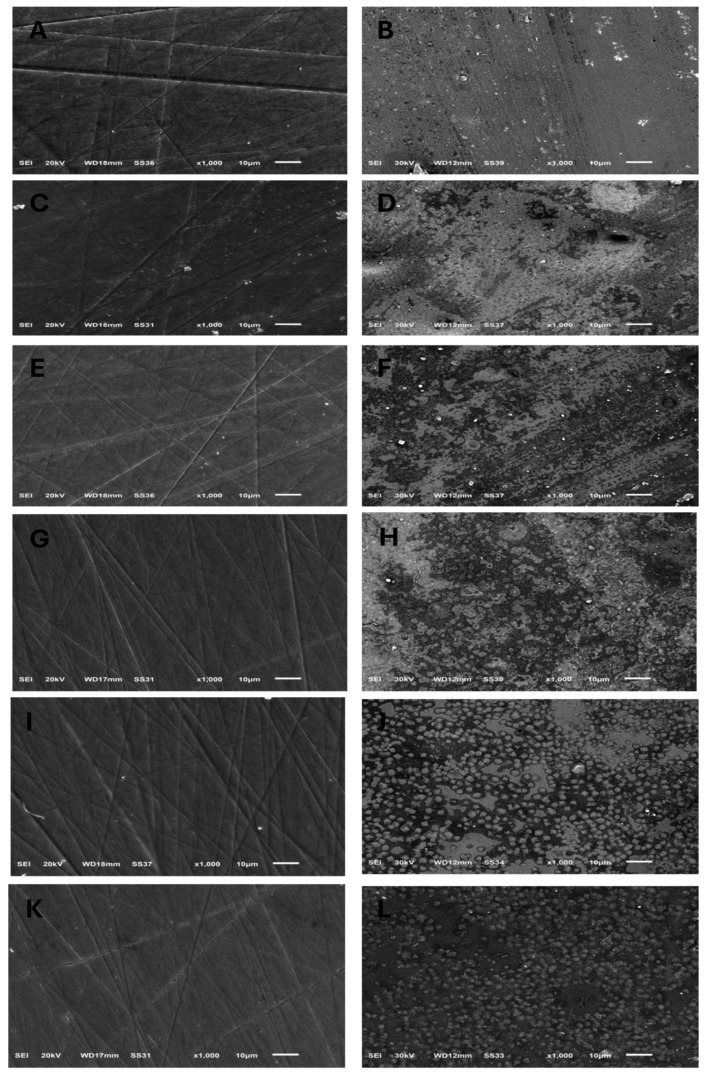
SEM images (×1000) showing surface topography of all tested ceramic groups before and after exposure to acidic challenge; (**A**,**B**) IPS e.max CAD, before and after acid exposure, respectively. (**C**,**D**) IPS e.max Press, before and after acid exposure, respectively. (**E**,**F**) GC LiSi Initial CAD, before and after acid exposure, respectively. (**G**,**H**) GC LiSi Initial Press, before and after acid exposure, respectively. (**I**,**J**) Cerec Tessera CAD, before and after acid exposure, respectively. (**K**,**L**) Celtra Duo Press, before and after acid exposure, respectively.

**Figure 2:**
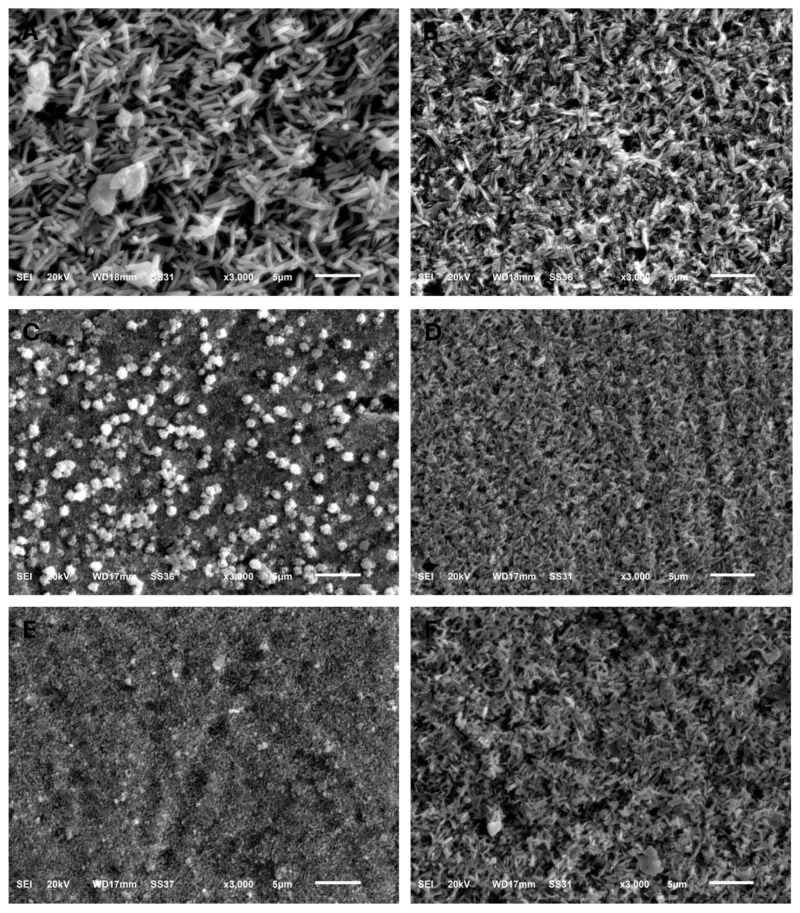
SEM images (×3000) showing the grain structure morphology of all groups. (**A**) IPS e.max CAD, (**B**) IPS e.max Press, (**C**) GC LiSi Initial CAD, (**D**) GC LiSi Initial Press, (**E**) CEREC Tessera CAD, (**F**) Celtra Duo Press.

The surface topography shown by SEM for specimens before acidic challenge exposure shows a flat surface with some minor grooves that may have resulted from finishing and polishing procedures (Figure 1A,C,E,G,I,K), which could then be filled with surface glaze. However, the surface topography of specimens after acidic challenge showed apparent darker spots, representing deeper areas than the lighter elevated surfaces of the IPS e.max types (Figure 1B,D), while larger darker areas representing deeper areas than the lighter elevated ones appeared on the GC LiSi Initials types (Figure 1F,H). Furthermore, clear and more defined surface crystals were noted in both the last images (Figure 1J,L) showing the Cerec Tessera CAD and Celtra Duo Press, respectively. Figure 2 shows the crystal morphology of ceramic groups. LDS materials underwent crystallization after being machined and had lower zirconia content (e.max CAD), revealing a microstructure with apparently large and elongated needle-like crystals. Its pressed type showed a similar crystal shape (Figure 2A,B). The GC LiSi Initial showed a similar shape as the pressed types, with more tendency towards an equiaxed structural appearance than its CAD type and apparently a much smaller crystal size (Figure 2C,D). CEREC Tessera apparently showed the smallest crystals with equiaxed shapes, while Celtra Duo Press showed an apparently smaller elongated crystalline structure (Figure 2E,F, respectively).

## 4. Discussion

The present study’s null hypotheses featured two suggestions that were both partially rejected. The first suggestion, according to the current results, shows the significant impact of glass ceramic composition and manufacture techniques on the surface microhardness in the tested groups, all before acidic exposure. However, either the material composition or manufacturing technique significantly affected BFS or Ra before acidic aging. On the other hand, after the acidic challenge, all variables showed no significant impact on the Ra. Additionally, the second suggestion was partially rejected, as all tested groups showed significantly lower surface microhardness after the acidic challenge. Nevertheless, acidic challenge did not significantly affect half the Ra values group or all BFS groups after acidic challenge exposure.

The flexural strength of glass ceramics is one important domain for their intra-oral performance. The results of the current study show the better mechanical performances of the pressed groups than their relative CAD groups both before and after acidic challenge exposure. IPS e.max showed higher BFS values for its pressed type, with a small difference from its CAD type before acidic challenge, while after the acidic challenge, the difference was significantly higher. This agrees with a recent work testing various commercial types of lithium disilicate (LDS) products, which identified the better mechanical performance of pressed types over CAD/CAM ones [11]. It could be noted that the flexural strength values of IPS e.max Press and CAD types were variable in previous studies. This could be attributed to the differences in composition and processing techniques. Moreover, the crystallization of IPS e.max Press is induced and controlled industrially. On the other hand, the crystallization of IPS e.max CAD occurs through two phases. Although the first lithium metasilicate (Li_2_ SiO_3_) crystalline phase is yielded and formulated by the manufacturer, the second phase is to be completed within the dental laboratory by heat treatment to complete the transformation of lithium metasilicate into lithium disilicate (Li_2_Si_2_O_5_) [24]. Pressed and CAD ingots used for IPS e.max showed no significant difference in their BFS with the same translucency [25]. The latter finding agrees with what was found in the current study of IPS e.max types, where the difference was small in favor of the pressed group before acidic challenge, wherein both groups had HT.

The same findings were obtained for the GC-LiSi types currently being assessed. However, the difference between GC-LiSi types was significantly greater after acidic challenge exposure. Additionally, the GC-LiSi Initial pressed type showed a statistically significant difference in BFS values from IPS e.max Press before and after acidic exposure. These former findings are in agreement with earlier results, wherein IPS e.max Press showed significantly higher flexural strength values than GC-LiSi Initial when exposed to a three-point bending strength test [19]. However, these results disagree with those of a former study [26] that assessed the biaxial flexural strengths of these pressed LDS ceramics. They reported no significant difference in flexural strength between both previous pressed types, although the microstructural images show larger-sized LDS crystals for IPS e.max (1.0–4.0 μm) than for GC-LiSi (1.0–1.5 μm). This could be attributed to the fact that flexural strength is related to more factors than just the crystal size, like crystalline phase distribution and crystalline-to-glassy matrix ratio [11].

Regarding the CAD types, IPS e.max and GC LiSi Initial, they showed a significant difference in BFS both before and after acidic challenge. In the latter case, IPS e.max CAD blanks are received as pre-crystallized, and require heat treatment for complete crystallization, which enriches the crystalline content [25]. After the crystallization had been completed, the glassy matrix was reported to show layered clusters. These clusters usually had different orientations in different directions, which may cause crack deflection when crack propagation within the glassy matrix occurs [27]. However, the GC-LiSi type undergoes no more crystallization after milling.

In a study undertaken in Boston University (2022) [22], Cerec Tessera, currently known as advanced lithium disilicate (ALDS), showed significantly higher BFS than the IPS e.max CAD when both had surface glazing, while these findings reversed in the case of ground or polished surfaces. The former findings do not agree with our findings, wherein ALDS showed significantly lower BFS than IPS e.max CAD, both before and after acidic aging. However, our findings agree with those of previous studies [21,28] reporting the significantly higher flexural strength values for IPS e.max CAD over ALDS. The ALDS material is a promising new glass ceramic category; however, the current studies are still insufficient for us to fully understand and compare this material with the currently existing glass ceramics. As claimed previously, ALDS consists of 90% LDS crystals and a 5% virgilite volume content [29]. However, this ALDS material showed the worst mechanical performance when compared to LDS and partially stabilized zirconia [30]. A recent review [31] stated that a smaller crystalline size in LDS-based ceramics compromised their mechanical performance—a concept that could be applicable to ALDS with smaller equiaxed crystalline sizes, as shown in the current microstructural images.

The zirconia-toughened lithium silicate (ZLSC) used in the current study (Celtra-Duo-Press) showed the second highest value of BFS, between the significantly higher values for e.max types and significantly lower values for other LDS GC-LiSi types. It showed no significant difference from ALDS. Previous studies that compared ZLSC with IPS e.max’s flexural strength showed wide variations in their results. According to previous in vitro works [32,33], which tested the BFS and three-point bending strength of both glass ceramic types, polished ZLSC (Suprinity) specimens showed significantly higher flexural strength than IPS e.max CAD. However, other studies [34,35] showed a significantly higher three-point bending strength for ZLSC (Suprinity) than IPS e.max CAD.

Additionally, there was a wide variation in the flexural strength values shown by previous studies, ranging from 289 to 415 MPa for IPS e.max CAD and from 230 to 510 MPa for ZLSC (Suprinity). Although VITA Suprinity and Celtra Duo do have the same chemical compositions, they go through different heat treatment processes. These treatments may cause slight differences in crystalline volume fractions. Celtra Duo exhibited a larger crystalline size and volume of Li_2_Si_2_O_5_ crystals in comparison to VITA Suprinity. This could explain its better mechanical performance in terms of hindering the crack propagation and improving the toughening mechanism within Celtra Duo [11]. However, the inferior flexural strength shown for ZLSC in the current study may also be attributed to the smaller crystalline size, as the material was reported to have nanometric Li_2_SiO_3_ granules and submicrometric Li_2_Si_2_O_5_ [36,37]. It appears that mechanical failure is more probable when the material’s microstructure falls into the micrometer, or more notably the nanometric, scale. The latter decreases the resistance to critical crack growth even in the presence of a larger fraction of crystalline phase [11]. However, it should be noted that the BFS mean values of all tested groups were higher than 300 MPa—this value, according to ISO 6872:2024, is the essential threshold for the construction of a monolithic crown or three-unit ridges up to the premolar area [37].

The current micro-hardness results are harmonious with the BFS results seen for all groups. The current findings again show higher surface hardness values for both types of IPS e.max than ALDS and ZLSC types, which had almost the same hardness values. These results agree with certain previous findings, wherein IPS e.max CAD showed a significantly higher surface microhardness than ZLSC (Celtra Duo), both in the crystallized state [21]. However, the current findings do not agree with those of previous work, wherein ALDS had the significantly highest surface hardness, followed by ZLSC and then IPS e.max CAD [38].

The former studies account for their findings via the addition of zirconia at 10%, which acts as a filler to enhance the mechanical performance of such a material [39]. However, in studying the role of zirconia in ZLSC, we should note that zirconia was not used as a crystalline phase in any of the commercial types to enrich the residual glass. On the contrary, there was a sign of zirconium segregation next to neighboring atoms during heat treatment in the form of amorphous zirconium nanoclusters. This was found to be stable throughout the heat treatment at 480–560 °C, but when the Li_2_SiO_3_ commenced crystallization at 560 °C, this began in network areas without zirconium, which contradicts the role of zirconia as a nucleating agent enhancing the crystallization process [40]. The present study shows the degradation of mechanical properties of these glass-based ceramics, in terms of BFS and microhardness, after the acidic challenge. This could possibly be attributed to the leaching of alkali ions, besides the disintegration of these ceramics glassy (Si–O–Si) networks by the acid [41].

The surface roughness (Ra) results of the current study agree with previous findings [21]. All studies stated the same findings of a significantly higher Ra for IPS e.max CAD than for ALDS and ZLSC. However, in a recent work, ZLSC showed a higher Ra than IPS e.max CAD [40], while ALDS still showed a significantly lower Ra than both ceramic types [38]. The current Ra findings could be explained with reference to the differences in surface roughness, which may depend on the individual properties of the materials [42,43], such as the types and sizes of the crystals together, with their distribution and quantity [16]. IPS e.max, according to the manufacturer, has a crystalline content of up to 70%, ranging from 60 to 70% for CAD and pressed types, respectively, while GC-LiSi Initial has a crystalline content of around 50–55% of its volume [11].

Additionally, the acidic challenge showed significant impacts in the form of increasing the surface roughness, namely, the Ra values, of both ALDS and ZLSC. There are extremely scarce studies examining the performances of both materials used in this study after exposure to simulated acidic challenge, to the knowledge of the authors. A previous study [44] showed a significant decrease in surface roughness for LDS and ZLSC (Suprinity) after exposure to simulated acidic challenge. The previous findings for ZLSC disagree with our findings. This disagreement could be attributed firstly to the shorter application time and lower HCl concentration used in a previous study, which was stated to be equivalent to 2 years of intra-oral exposure [45]. The current study used a 37% HCl concentration and 1.2 pH for 24 h to investigate the long-term effects of the acidic challenge on different types of LDS ceramics. The latter period of storage could be equivalent to a few years in the patient’s mouth [46]. Furthermore, they explained their findings as relating to the ZLSC microstructure used [32,47]. LSC crystals are more prone to dissolution, yielding a smaller crystalline structure of 500–700 nm, aside from the appearance of nanosized pores on their SEM. However, aside from the current exposure conditions equivalent to a longer intra-oral period, the current SEM images show much broader darker and lighter surface areas, although these were recorded with greater magnification than previously used [44]. The current appearance could be attributed to the longer period of acidic exposure causing uneven dissolution in surface areas, and thus a rougher surface after acidic exposure. This might also explain the similar appearance on the SEM images of ALDS after acidic challenge exposure.

On the contrary, a recent study [16] has agreed with the roughness values derived for ZLSC (Suprinity) after a longer period of acidic challenge equivalent to 10 years in the intra-oral environment, for which they used atomic force microscopy rather than an optical profilometer. As the relevant results are scarce, future studies are recommended on the impacts of a longer acidic challenge of these two materials, where surface roughness evaluation is to be undertaken in regular intervals. This will allow for monitoring the changes in all roughness parameters (Ra, Rt and Rz) and not only Ra, which is a limitation of the current study, yielding a better understanding of the materials’ surface performance. The roughness values of all groups after exposure to acidic challenge showed no significant differences, with the reported values all being above 0.2 μm—the threshold that favors microbial adhesion [48]. On the other hand, before acidic challenge, ALDS and ZLSC showed Ra values of less than 0.2 μm, which reflects the better intra-oral biological response.

The current in vitro model shows a limitation of this study, as these models cannot consider the role of the complex oral environment [49]. Another limitation of the current study is that it tested only one acidic challenge, mostly simulating the gastric challenge condition. Other acidic media, like green mango juice, pineapple juice and cola, all had more significant impacts on Ra and surface micro-hardness than HCl [50]. Further studies are needed to test the impacts of the studied degradation in ceramics’ surface properties on their interaction with natural opposite dentition. Additionally, further investigations on the mechanical fatigue survival behavior of such acid-aged ceramics are needed.

## 5. Conclusions

Based on the results of the current study, the following conclusions can be drawn:

- Pressed LDS-based glass ceramics show better mechanical performance than milled ones;
- IPS e.max showed a higher flexural strength and surface microhardness than advanced LDS and zirconia-toughened LSC;
- The acidic challenge showed strong impact on the BFS and surface microhardness of the tested ceramics;
- The Ra values of most studied ceramics were higher than the recommended values, and would favor dental plaque accumulation.

## Figures and Tables

**Table 1:** Glass ceramic materials used in the current study.

Type	Commercial Name	Constituents	Manufacturer	Patch No.
a. Pressable glass ceramics				
Lithium disilicate (Li_2_Si_2_O_5_)	IPS e.max^®^ Press	SiO_2_ (57–80%), Li_2_O (11–19%), K_2_O (0–13%), P_2_O_5_ (0–11%), ZrO_2_ (0–8%), ZnO (0–8%), Coloring Oxides (0–12%)	Ivoclar Vivadent, Schaan, Liechtenstein	Z036XN
	GC Initial^TM^ LiSi Press	SiO_2_, Al_2_O_3_, LiO_2_, K_2_O, P_2_O_5_, ZrO_2_, Na_2_O, K_2_O [19]	GC, Tokyo, Japan	170306A
Zirconia-reinforced lithium silicate (Li_2_SiO_3_/Li_2_Si_2_O_5_)	Celtra ^®^ Duo press	SiO_2_ (58%), ZrO_2_ (10%), P_2_O_5_, Al_2_O_3_, Li_2_O, and ZnO	Dentsply Sirona, NC, USA	16010742
b. CAD/CAM glass ceramics				
Lithium disilicate (Li_2_Si_2_O_5_)	IPS e.max^®^ CAD	SiO_2_ (57–80%), Li_2_O (11–19%), K_2_O (0–13%), P_2_O_5_ (0–11%), ZrO_2_ (0–8%), ZnO (0–8%), Coloring Oxides (0–12%).	Ivoclar Vivadent, Schaan, Liechtenstein	Z01SBK
	GC Initial LiSi Block	SiO_2_ (81%), P_2_O_5_ (8.1%), K_2_O (5.9%), Al_2_O_3_ (3.8%), TiO_2_ (0.5%), CeO_2_ (0.6%) [20]	GC, Tokyo, Japan	2112221
	CEREC Tessera	Li_2_Si_2_O_5_: (90%) Li_3_PO_4_: (5%) Li_0.5_Al_0.5_Si_2.5_O_6_ (virgilite): (5%)	Dentsply Sirona, York, PA, USA	16013117

**Table 2:** Parameters for different pressable glass ceramic processing technique.

Material	Stand by Temperature (°C)		Closing Time (min)	Heating Rate (°C/min)	Holding Temperature (°C)	Holding Time (min)	Vacuum On/Off (°C)	Long-Term Cooling (°C)
IPS e.max^®^ Press	Pressing	700	__	60	920	15:00	500/920	__
	Glazing	403	6:00	60	770	1:00–2:00	450/769	500
GC-LiSi Press	Pressing	700	__	60	898	25:00	__	__
	Glazing	480	2:00	45	810	1:00	__	__
Celtra Duo Press	Pressing	700	3:00	40	860	30:00	__	__
	Glazing	400	2:00	55	760	2:00	__	__

**Table 3:** Parameters for different CAD/CAM glass ceramic processing techniques.

Materials		Stand-by Temperature (°C)	Closing Time (min)	Heating Rate (°C/min)	Heating Rate 2 (°C/min)	Holding Temperature (°C)	Holding Temperature 2 (°C)	Holding Time (min)	Holding Time 2 (min)	Vacuum On/Off (°C)	Long-Term Cooling (°C)
IPS e.max^®^ CAD	Crystallization	403	6:00	60	30	770	850	0:10	10:00	770/850	700
	Glazing	403	6:00	60	__	725	__	1:00	__	450/724	__
GC Initial LiSi Block	Crystallization	Not Required									
	Glazing	480	4:00	45	__	740	__	1:00	__	No	__
CEREC Tessera	Crystalliz-ation	Not Required									
	Glazing	400	3:30	60	__	760	__	2:00	__	__	__

**Table 4:** Two-way ANOVA showing the impacts of different variables and the interactions between them on the BFS, surface micro-hardness, and surface roughness (Ra) for all groups before acidic aging.

Source	BFS		Surface Microhardness		Surface Roughness (Ra)	
	F	*p*-Value	F	*p*-Value	F	*p*-Value
Corrected model	4.768	0.012 *	34.718	0.000 *	13.983	0.000 *
Intercept	2.257 × 10^3^	0.000 *	2.256 × 10^5^	0.000 *	986.899	0.000 *
Composition	11.625	0.002 *	48.252	0.000 *	3.199	0.083
manufacture	0.577	0.462	25.147	0.000 *	18.321	0.000 *
Composition X manufacture	0.007	0.993	24.374	0.000 *	13.240	0.000 *

* means significant difference at *p*-value ≤0.05.

**Table 5:** Two-way ANOVA showing the impacts of different variables and the interaction between them on the BFS, surface micro-hardness and surface roughness (Ra) for all groups after acidic aging.

Source	BFS		Surface Microhardness		Surface Roughness (Ra)	
	F	*p*-Value	F	*p*-Value	F	*p*-Value
Corrected model	6.08	0.01 *	219.39	<0.01 *	1.94	0.10
Intercept	2.39 × 10^3^	<0.01 *	1.93 × 10^5^	<0.01 *	1.26 × 10^3^	<0.01 *
Composition	12.56	<0.01 *	149.70	<0.01 *	0.07	0.79
Manufacture method	4.30	0.06	157.53	<0.01 *	2.37	0.09
Composition X manufacture method	0.49	0.63	309.70	<0.01 *	2.14	0.12

* means significant difference at *p*-value ≤0.05.

**Table 6:** Means and standard deviations (SD) and Student *t*-test for biaxial flexural strength (BFS) in MPa before and after acidic aging.

Groups	Means ± SD Before Aging	Means ± SD Before Aging	Student *t*-Test Before and After Aging
IPS e.max CAD	400.10 ± 38 **^a^**	366.86 ± 28 **^AB^**	t = 1.38 and *p*-value = 0.24
IPS e.max Press	409.95 ± 27 **^a^**	401.05 ± 20 **^A^**	t = 0.41 and *p*-value = 0.70
GC LiSi CAD	308.56 ± 19 **^c^**	282.59 ± 19 **^C^**	t = 1.47 and *p*-value = 0.22
GC LiSi Press	322.64 ± 28 **^ab^**	311.67 ± 23 **^C^**	t = 0.47 and *p*-value = 0.66
Cerec Tessera CAD	367.65 ± 40 **^ab^**	317.89 ± 40 **^C^**	t = 1.43 and *p*-value = 0.23
Celtra Duo Press	378.71 ± 38 **^ab^**	329.26 ± 32 **^AB^**	t = 1.60 and *p*-value = 0.19
*p*-value	*p*-value = 0.01 *	*p*-value = 0.01 *	

* means significant difference at *p*-value ≤0.05. Letters are for Tukey’s post-hoc test for each group before and after aging, separately.

**Table 7:** Means and standard deviations (SD) and Student *t*-test for surface micro-hardness (gf/μm^2^) before and after acidic aging.

Groups	Means ± SD Before Aging	Means ± SD After Aging	Student *t*-Test
IPS e.max CAD	533.37 ± 1.76 ^ab^	497.17 ± 4.25 ^BC^	t = −5.98 & *p*-value < 0.01 *
IPS e.max Press	541.13 ± 1.40 ^a^	499.83 ± 5.21 ^BC^	t = 13.26 & *p*-value < 0.01 *
GC LiSi CAD	492.77 ± 3.67 ^d^	485.77 ± 2.32 ^C^	t = −2.80 & *p*-value = 0.05 *
GC LiSi Press	520.03 ± 3.35 ^bc^	481.70 ± 9.61 ^D^	t = 6.52 & *p*-value < 0.01 *
Cerec Tessera CAD	527.03 ± 1.59 ^ab^	383.00 ± 5.63 ^E^	t = 42.60 & *p*-value < 0.01 *
Celtra Duo Press	527.67 ± 2.50 ^ab^	509.83 ± 9.42 ^B^	t = −3.17 & *p*-value = 0.03*
*p*-value	*p*-value < 0.01 *	*p*-value < 0.01 *	

* means significant difference at *p*-value ≤0.05. Letters are for Tukey’s post-hoc test for each group before and after aging, separately.

**Table 8:** Means and standard deviations (SD) and Student *t*-test for Ra (μm) before and after acidic aging.

Groups	Means ± SD Before Aging	Means ± SD Before Aging	Student *t*-Test
IPS e.max CAD	0.34 ± 0.04 ^a^	0.26 ± 0.05	t = 2.68 and *p*-value = 0.03 *
IPS e.max Press	0.36 ± 0.10 ^a^	0.32 ± 0.04	t = 0.00 and *p*-value = 1.00
GC LiSi CAD	0.42 ± 0.06 ^a^	0.38 ± 0.03	t = 1.42 and *p*-value = 0.19
GC LiSi Press	0.35 ± 0.01 ^a^	0.31 ± 0.07	t = 1.95 and *p*-value = 0.09
Cerec Tessera CAD	0.19 ± 0.07 ^b^	0.36 ± 0.07	t = −3.74 and *p*-value = 0.01 *
Celtra Duo Press	0.18 ± 0.02 ^b^	0.33 ± 0.09	t = −3.87 and *p*-value = 0.01 *
*p*-value	*p*-value < 0.01 *	*p*-value = 0.12	

* means significant difference at *p*-value ≤0.05. Letters are for Tukey’s post-hoc test for each group before and after aging, separately.

## Data Availability

The data used for study are available from the corresponding author.
